# Performance Evaluation of an Ultrasonic Imaging System Using Tissue-Mimicking Phantoms for Quality Assurance

**DOI:** 10.3390/biomimetics7030130

**Published:** 2022-09-11

**Authors:** Ammar A. Oglat

**Affiliations:** Department of Medical Imaging, Faculty of Applied Medical Sciences, The Hashemite University, Zarqa 13133, Jordan; ammara@hu.edu.jo; Tel.: +962-796-311-835

**Keywords:** calibration, axial and lateral resolution, focal zone, transducers, diagnostic ultrasound, gel tissue-mimicking phantoms, ultrasound quality assurance

## Abstract

Diagnostic ultrasound or sonography is an image that can provide valuable information for diagnosing and treating a variety of diseases and conditions. The aim of this research study is to examine the performance and accuracy of the ultrasonic imaging system for the guarantee of diagnosis quality assurance, and to adjust the penetration settings to minimize the time of repeat scans and maintenance duration during research experiments. Measurements in this experiment included the resolution (axial and lateral) and focal zones. Moreover, the evaluation was done by completing all the measurements at different depths on a multipurpose phantom model 539. The phantom was bought from the market and was not fabricated by the author. The measurements were achieved by applying two different transducers: curved and linear (flat). The ultrasound images were obtained and tested by using calipers (electronic), and the estimations and observations were read by using all the taken measurements and images. As a result, because the phantom depths were different, the penetration settings were different too, indicating that the depth impacted the penetrations of the created ultrasound image. Moreover, after the comparison of the recorded measurements and results, it was found that all measurements were within the accepted (standard) value and that the true value was specified by the production of the phantom.

## 1. Introduction

Medical sonography is an imaging technique that employs the great-frequency of sound energies (more than 20 KHz) to create images of organs (internally) and anatomy within the human body [1,2,3,4,5,6,7]. In general, medical doctors use it to view different parts of the body, such as the heart, vessels, kidneys, liver, and other internal organs [8,9,10,11,12,13]. In addition, they use ultrasound devices to show the fetus during the pregnancy period [14,15,16]. The image features can be provided with high quality for both diagnosis and treatment of diseases [17].

The two types of sonar devices are outside devices and inside devices. Nearly all ultrasound scans are made via a sonar tool on the external side of the patient’s body parts. Otherwise, an unspecified number of ultrasound scans involve operating a tool on the internal side of the patient’s body parts [18].

When an ultrasound measurement is performed, a tool called a transducer or probe will be applied over part of your body. This tool (transducer) transmits sound energies which later recirculate off the tissues on the internal side of the patient’s body. Furthermore, it also receives the energy that recoils back (reflected from the tissue) [19,20,21,22,23,24,25].

The ultrasound images are created by ultrasonic sound waves; these waves are longitudinal and produce particles that wiggle front and back, which then gives a compression group (particles are close to each other) and a rarefaction group (particles are farthest from each other). The structures of the body can be seen in ultrasound images during the movement of internal organs and the flow of blood through vessels. This is due to the ability of the machine to capture data in real-time [21,26,27].

The most common probes used in medical ultrasound are curved and flat (linear). However, the frequencies of curved s range from 2 to 5 MHz to reflect a range of humans from slender to obese. This is an adjustment between flat and sector devices. When the distance from the probe increases, the density of the scan lines decreases [21,24,28,29,30,31,32,33,34]. Linear transducers, on the other hand, are sometimes used at high frequencies of 7 MHz to produce parallel sound energies; additionally, the resulting image is rectangular. The image width and the scan line quantities are similar at whole tissue levels. This will thus lead to the feature of a perfect resolution (near field) [9,10,31,35,36,37].

Furthermore, this is a straightforward, all-inclusive method of assessing imaging applications across nearly all clinical frequencies (from 2 to 20 MHz). For the distance measurements, the fabrication of the phantom is done with a set of single filament line points while the tissue simulating goal forms diverge in sizes and shapes. Because of the acoustic identity of the background item and the goal forms, artifacts produced by deformation and enhancement have been removed. Six gray-scale goals from +15 to −15 dB were measured to evaluate the tools shown for both energetic range and gray-scale handling achievement [38,39,40].

The main objective of this study is to examine the performance and accuracy of the ultrasonic imaging device by performing some measurements (the resolution (axial and lateral) and focal zone) on the tissue-simulating phantom.

## 2. Methods

**Figure 1:**
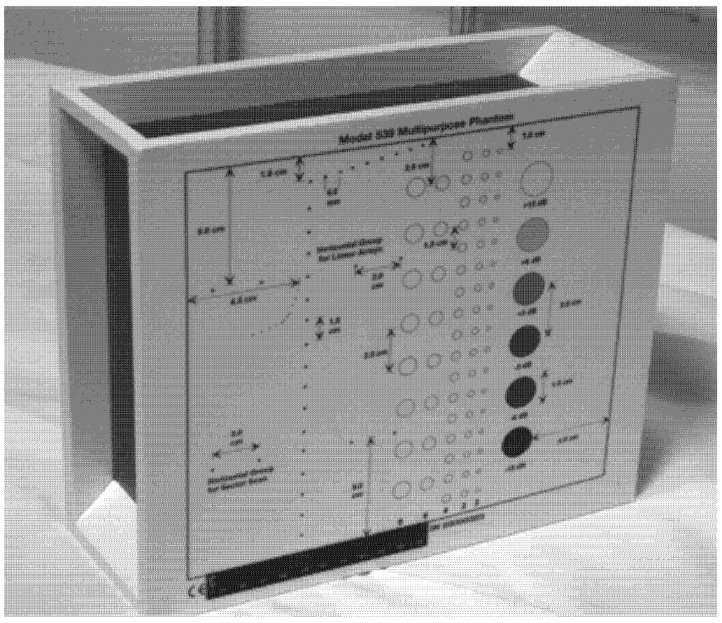
Multipurpose phantom model 539.

In the current experiment, tissue-simulating phantom quality assurance was applied to examine the performance and accuracy of the ultrasound imaging device (Figure 1). The phantom simulates the acoustic features of real tissue and supports the measurement of structures in an encouraging environment. The imaging system can detect any alterations in performance that happen during the ordinary lifetime of imaging system parts. Thus, routine examination control is able to minimize the time of repeat scans and maintenance duration.

The tissue-mimicking phantom and diagnostic imaging systems are calibrated at room temperature (23 °C). Therefore, measurements need to be made at the normal temperature. The multipurpose phantom (phantom model 539), on the other hand, is used to hold the test and is designed to provide the operator with an all-inclusive way of examining the capability of the linear array, annular array, sector, phased array, and diagnostic technique. Several significant ultrasound device functions must be understood while conducting this research study. These properties are essentially global to all medical ultrasound instruments.

The model 539 multipurpose phantom is an easy, comprehensive means of evaluating imaging systems over the full range of clinical imaging frequencies (2 MHz to 18 MHz). This phantom is designed with a combination of monofilament line targets for distance measurements and tissue mimicking target structures of varying sizes and contrasts. Cystic-like target structures are positioned in-line vertically, thereby permitting an entire target group to be displayed in one view. Due to the acoustic similarity of the background material and the target structures, artifacts caused by distortion, shadowing or enhancement are eliminated. Six gray scale targets ranging in contrast from +15 to −15 dB are provided to evaluate the system’s displayed dynamic range and gray-scale processing performance. All ATS urethane phantoms are guaranteed for the useful life of the phantom, defined as 10 years.

A clinical ultrasound scanner (HI) was connected with probes to collect the information and data from the phantom. The probes were mounted on a movable probe stand, and the phantom was placed on a suitable flat table. The probe was constant at a required angle (diagonal) and lowered nearly to the surface of the phantom to prevent pressing the probe into the TMM. In in-vitro studies, the cross-sectional and longitudinal images are taken as part of the evaluation process of TMM to diagnose and check the validity of TMM for other applications such as obstructions.

Two different probes (curved and linear) were used in this study. The curved probe has frequencies of 2–5 MHz to allow for a range of patients from obese to slender. This is a compromise between linear and sector scanners. The density of the scan lines decreases with increasing distance from the transducer. Further, the linear probe is often used at high frequencies of 7 MHz. This produces sound waves parallel to each other and produces a rectangular image. The width of the image and the number of scan lines are the same at all tissue levels. This has the advantage of good near-field resolution.

The first experiment was to measure the axial and lateral resolution of the ultrasound system. The tools used in this section are ultrasound machines with curve and flat transducers, coupling viscous gel, phantom 1 and phantom 2 (model 539). The step was started by putting the phantom on a washed suit surface with scanning surface 1. A sufficient quantity of viscous gel was placed on the scan surface. To set standard values for the scanning of a normal liver, tool adjustments such as time gain compensation (TGC), output, and so on were used. The lowest point (bottom) of the phantom was pictured, and the gain unit was modified. The screen display was frozen to take measurements. The image was determined whether all of the line points were obviously represented as separate points or not. Finally, the observations were documented on the quality assurance record. However, the above procedures were repeated for the remaining three depths by using scanning surfaces 2, 3, and 4 and for both types of transducers.

The second experiment was to measure the focal zone of the ultrasound imaging system. Ultrasound techniques with flat and curved transducers, viscous couple gel, phantom 1 and phantom 2 were applied in this research. The first procedure began by cleaning the phantom, creating a smooth surface with scanning surface 1, applying an adequate amount of couple gel to the scan face, adjusting the output of the equipment, and using TGC to set standard values for liver scanning. The end of the phantom was scanned and the unit of gain tuning was set. The transducer was placed through the vertical collection of line points until an obvious image was acquired. The phantom was scanned with several different focal zone settings for a variable focused transducer. The line linking the bottom of the echoes retrieved from the line points (both sides) was drawn on the hard copy of the image, and the narrowest portion was located. The beam’s width and depth were measured at this point. Finally, measurements of the focal zone depth and width were documented on a quality assurance register.

## 3. Results and Discussions

Standardized image quality test methods are a critical component of product development, quality assurance, and regulatory decision-making, all of which often require direct performance comparison between design configurations or finalized device products.

An axial resolution is the lowest separation between parts that the ultrasound energy can recognize parallel to the echo track. In addition, it is most influenced by transducer frequency. It was applied to measure the capacity to check the dots (wires) in the phantom model separately and for its ability to see cysts and carefully measure them. While lateral resolution is the minimum separation from one point to another point or clinically different tissue, the ultrasound energy can differentiate at a level perpendicular to the ultrasound energy. However, the table below (Table 1) shows the real distance for axial and lateral resolution for both curves and flat or linear probes.

Representative ultrasound and photoacoustic images acquired in solid filament array phantoms are presented for the transducer. The results of lateral and axial resolution for phantom 1, tested on surface 1, indicated that there was no problem measuring the distance for axial and lateral resolutions by using a curve probe (Table 2). However, the measurement can only be made on a 9.0 cm setting by using a flat or linear probe. The axial and lateral resolutions cannot be measured at 5.0, 6.0, and 7.0 cm depths (Table 3). This is because it cannot penetrate deep enough to capture the kidney-mimicking image on the phantom. As we know from the previous test, the kidney-mimicking image was located approximately 6.0 cm or 60.0 mm deep on the phantom 1 from surface 1.

### 3.1. Phantom 1

For surface 2, all measurements were done by using both a curve and a flat probe. When the results of the distance that had been recorded for axial and lateral resolutions were compared with the distances given by the manufacturer on the probe, the difference is small, with an average of 0.2 mm (Table 4 and Table 5).

For surface 3, the measurements cannot be achieved at 14.0 and 16.0 cm when using a curve probe (Appendix A Table A1 and Table A2). This is because the kidney-mimicking image was found on surface 3 at a depth beyond, and can only be measured at 18.0 and 24.0 cm. Due to the distance where the image is located from the surface, the measurement cannot be achieved using a linear probe. This is because of the low penetration that this this probe is capable of.

This also happened on surface 4, (Appendix A Table A3 and Table A4); the measurements could not be obtained using a linear probe because of the deep location of the kidney. Mimicking the image, however, can only be done by using a linear probe. All the measurements recorded for both surfaces 3 and 4 were only slightly different from the real distance.

Regarding phantom 2, only linear probes were applied. This is because phantom 2 is shorter than phantom 1, and the linear probe had a lower penetration setting compared to the curve probe. Therefore, a linear probe is more suitable for the phantom. Based on the results recorded on phantom 2, axial and lateral resolution measurements were still under an acceptable value since the percentage difference between the actual distances was less than 2% (Table 6).

Vertical resolution did not depend significantly on target depth in the phantom. However, the vertical resolution spot size increased with depth in the phantom. This effect is caused by the phantom’s nonlinear (with frequency) acoustic attenuation coefficient, which decreases bandwidth with propagation depth [41]. However, lateral resolution was similar for ultrasound images. This is because plane-wave transmission was used for ultrasound imaging, and the lateral resolution was therefore completely determined by receive beamforming, which was true for both ultrasound images.

### 3.2. Phantom 2

On the other hand, a focal zone in ultrasonography is defined as the distance along the beam axis of a focused transducer assembly from the point where the beam area first becomes equal to four times the focal area. Put simply, the focal zone can also be described as the area in the ultrasound beam that has the smallest beam diameter and where a user will get the best side-to-side or lateral resolution. It is the area of the transducer where the sound beam is most sharply focused and where the area under examination will give the best image. However, focal zones allow multiple focus points. As the number of focal zones increases, the frame rate will decrease, and the image will slowly be refreshed. This offers the best lateral resolution [42].

The results demonstrate why there is a difference at every depth. As depth increases, the focal zone changes to the lower point of the dots. Hence, penetration increases with depth, and therefore the focal zone changes to the lower point. In this part of the test, the focal zone along the vertical line was found. However, this cannot be done for the 5.0 and 6.0 cm settings of depth by linear probes. This is due to penetration by using depth (Appendix B Table A5, Table A6 and Table A7). In addition, as is known, the focal zone has the best lateral or side-to-side resolution. 

## 4. Conclusions

The phantom was obtained from the market for research studies which consisted of a robustly evaluated suite of phantom-based test methods for characterizing key aspects of ultrasound image quality in an objective, quantitative, and reproducible manner. The results display excellent accuracy of the value specified by the producer for this machine. Although there was a little difference between the result and the real distance, this variation is within the range of acceptable values. This variation occurred due to several errors, such as operator error when taking measurements. Thus, depth adjustment in the medical ultrasound machine influences penetration and then the image. In other words, depth increases when penetration is increased too. In general, axial and lateral resolutions are significant factors to ensure that the precision of distance tests is correct. In conclusion, the ultrasound technique is in perfect condition and can be applied to a clinical target without any misdiagnosis. Recurrent calibration tests and quality control should be achieved to ensure that the ultrasound tool is in valid condition.

## Figures and Tables

**Table 1:** The actual distance for axial and lateral resolution.

Axial Resolution (mm)	Lateral Resolution (mm)
D1 = 5.0 D2 = 4.0 D3 = 3.0 D4 = 2.0	D1 = 5.0 D2 = 4.0 D3 = 3.0 D4 = 2.0

**Table 2:** The distance for axial and lateral resolution in mm for depth of 14.0, 16.0, 18.0 and 24.0 cm; depth for curve probe at surface 1.

Depth (cm)	Distance (mm)	
	Axial Resolution	Lateral Resolution
14.0	D1 = 4.6 D2 = 3.9 D3 = 3.2 D4 = 1.9	D1 = 5.1 D2 = 4.1
16.0	D1 = 5.2 D2 = 3.9 D3 = 2.9	D1 = 5.0 D2 = 4.0
18.0	D1 = 4.9 D2 = 4.0 D3 = 3.1	D1 = 5.0 D2 = 3.8
24.0	D1 = 4.7 D2 = 4.1 D3 = 3.2	D1 = 5.0 D2 = 4.2

**Table 3:** The distance for axial and lateral resolution in mm for depth of 5.0, 6.0, 7.0 and 9.0 cm; depth for flat probe at surface 1.

Depth (cm)	Diagram	Distance (mm)
5.0	The horizontal distance cannot be measured for the image of this depth	-
6.0	The horizontal distance cannot be measured for the image of this depth	-
7.0	The horizontal distance cannot be measured for the image of this depth	-
9.0	Axial resolution 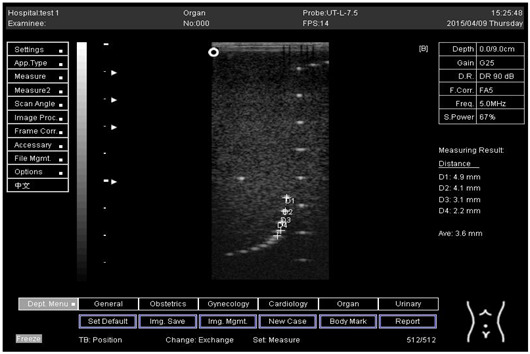	D1 = 4.9 D2 = 4.1 D3 = 3.1 D4 = 2.2
	Lateral resolution 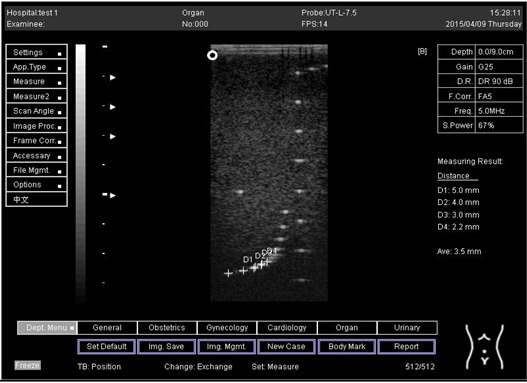	D1 = 5.0 D2 = 4.0 D3 = 3.0 D4 = 2.2

**Table 4:** The distance for axial and lateral resolution in mm for depth of 14.0, 16.0, 18.0 and 24.0 cm; depth for curve probe at surface 2.

Depth (cm)	Distance (mm)	
	Axial Resolution	Lateral Resolution
14.0	D1 = 5.0 D2 = 4.0 D3 = 2.9	D1 = 5.0 D2 = 4.1
16.0	D1 = 4.8 D2 = 4.0 D3 = 3.0	D1 = 4.8 D2 = 3.9
18.0	D1 = 5.0 D2 = 4.0 D3 = 3.0	D1 = 5.0 D2 = 4.0
24.0	D1 = 5.0 D2 = 4.1 D3 = 2.9	D1 = 4.7 D2 = 3.9

**Table 5:** The distance for axial and lateral resolution in mm for depth of 5.0, 6.0, 7.0 and 9.0 cm; depth for flat probe at surface 2.

Depth (cm)	Diagram	Distance (mm)
5.0	Axial resolution 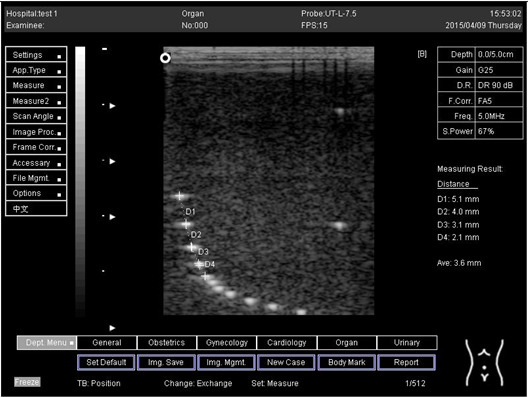	D1 = 5.1 D2 = 4.0 D3 = 3.1 D4 = 2.1
5.0	Lateral resolution 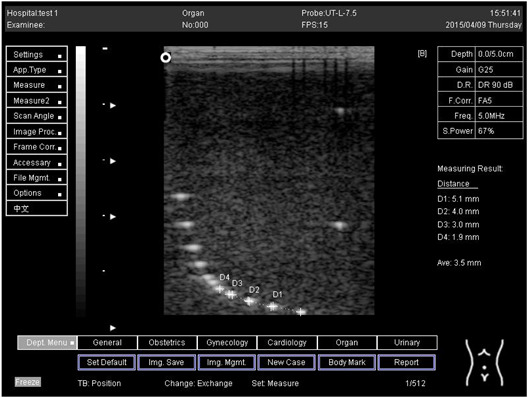	D1 = 5.1 D2 = 4.0 D3 = 3.0 D4 = 1.9
6.0	Axial resolution 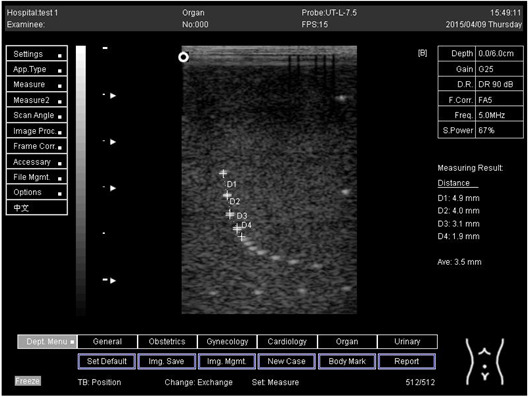	D1 = 4.9 D2 = 4.0 D3 = 3.1 D4 = 1.9
	Lateral resolution 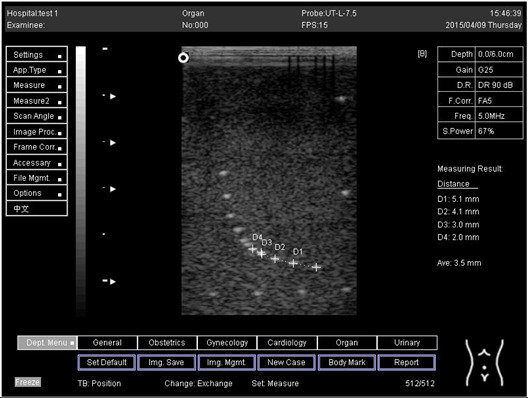	D1 = 5.1 D2 = 4.1 D3 = 3.0 D4 = 2.0
7.0	Axial resolution 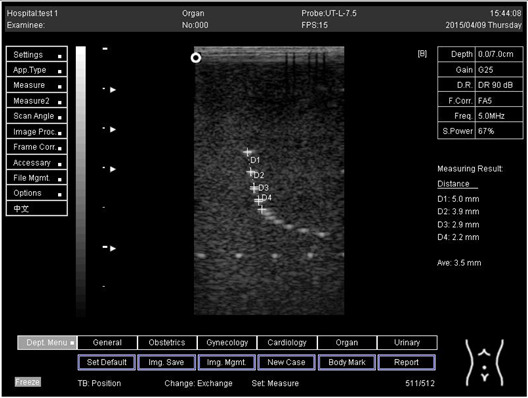	D1 = 5.0 D2 = 3.9 D3 = 2.9 D4 = 2.2
	Lateral resolution 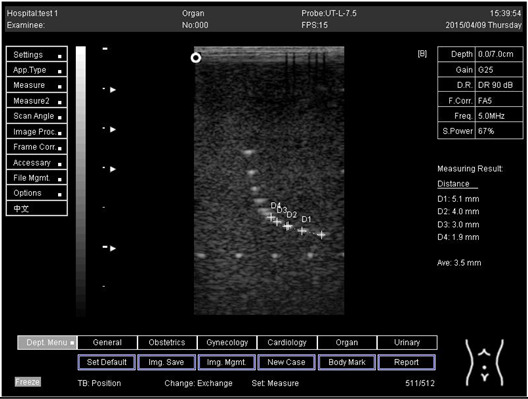	D1 = 5.1 D2 = 4.0 D3 = 3.0 D4 = 1.9
9.0	Axial resolution 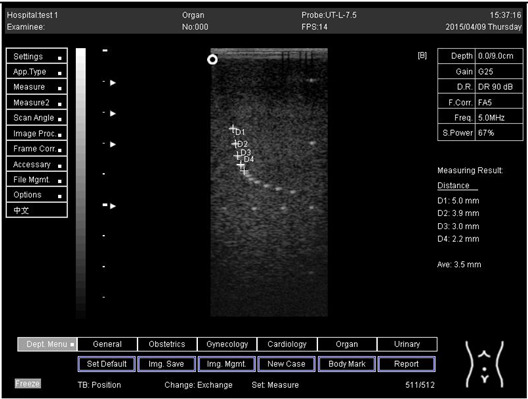	D1 = 5.0 D2 = 3.9 D3 = 3.0 D4 = 2.2
	Lateral resolution 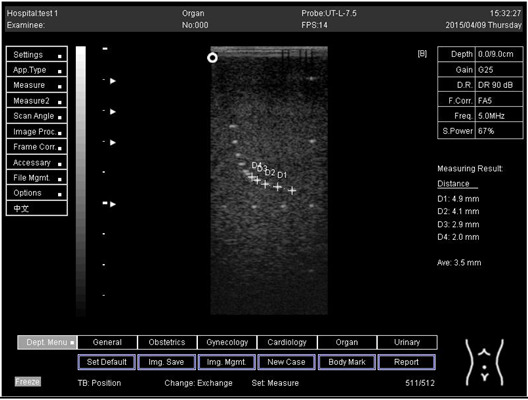	D1 = 4.9 D2 = 4.1 D3 = 2.9 D4 = 2.0

**Table 6:** The distance for axial and lateral resolution in mm for depth of 5.0, 6.0, 7.0 and 9.0 cm; depth for flat probe on phantom 2.

Depth (cm)	Diagram	Distance (mm)
5.0	Axial resolution 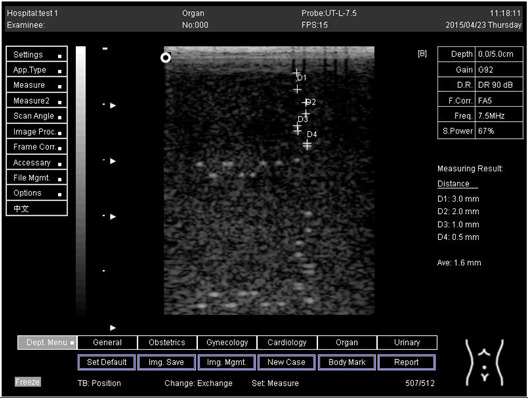	D1 = 3.0 D2 = 2.0 D3 = 1.0 D4 = 0.5
5.0	Lateral resolution 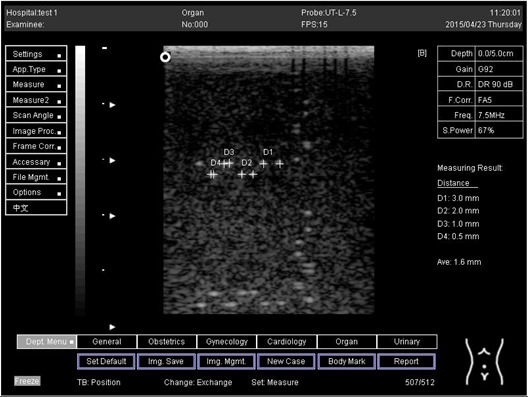	D1 = 3.0 D2 = 2.0 D3 = 1.0 D4 = 0.5
6.0	Axial resolution 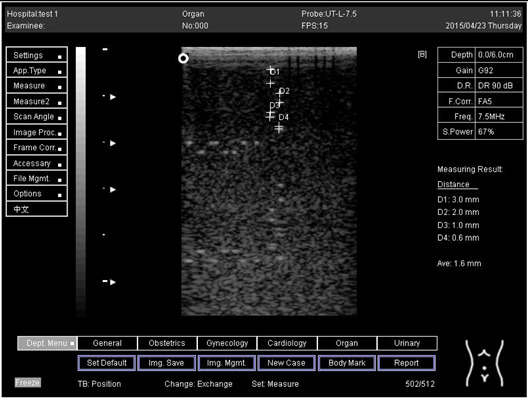	D1 = 3.0 D2 = 2.0 D3 = 1.0 D4 = 0.6
6.0	Lateral resolution 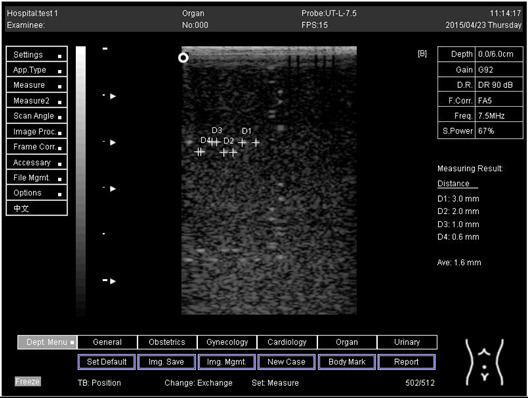	D1 = 3.0 D2 = 2.0 D3 = 1.0 D4 = 0.6
7.0	Axial resolution 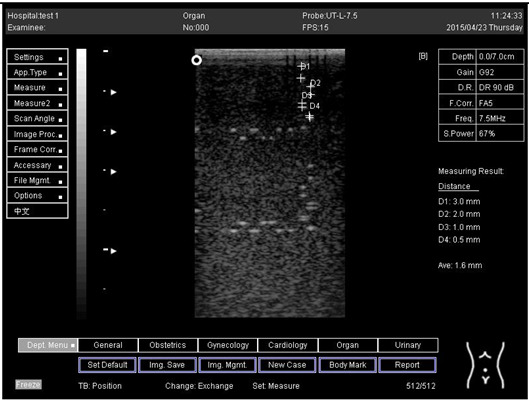	D1 = 3.0 D2 = 2.0 D3 = 1.0 D4 = 0.5
7.0	Lateral resolution 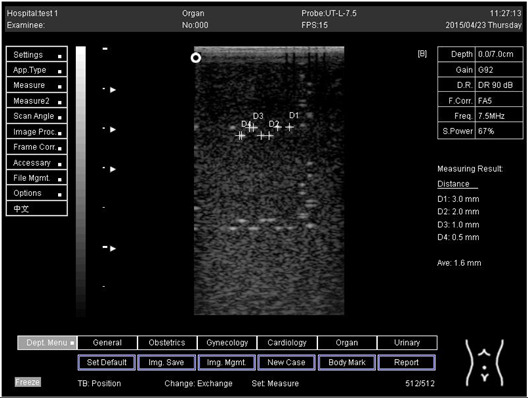	D1 = 3.0 D2 = 2.0 D3 = 1.0 D4 = 0.5
9.0	Axial resolution 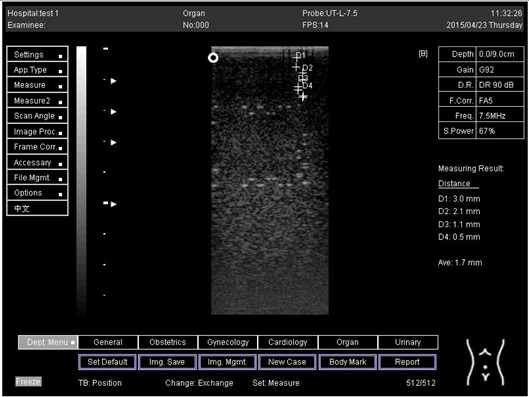	D1 = 3.0 D2 = 2.1 D3 = 1.1 D4 = 0.5
9.0	Lateral resolution 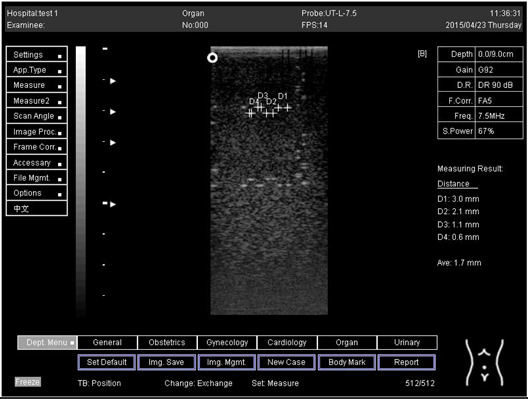	D1 = 3.0 D2 = 2.1 D3 = 1.1 D4 = 0.6

## Data Availability

Not applicable.

## Appendix A

**Table A1:** The distance for axial and lateral resolution in mm for depth of 14.0, 16.0, 18.0 and 24.0 cm depth for curve probe at surface 3.

Depth (cm)	Diagram	Distance (mm)
14.0	The horizontal distance cannot be measured for the image of this depth	-
16.0	The horizontal distance cannot be measured for the image of this depth	-
18.0	Axial resolution	D1 = 5.0
18.0	Lateral resolution	D1 = 5.0 D2 = 4.1
24.0	Axial resolution	D1 = 4.8 D2 = 4.1
24.0	Lateral resolution	D1 = 5.3

**Table A2:** The distance for axial and lateral resolution in mm for depth of 5.0, 6.0, 7.0 and 9.0 cm depth for flat probe at surface 3.

Depth (cm)	Diagram	Distance (mm)
5.0	The axial and lateral resolution cannot be measured for the image of this depth	-
6.0		-
7.0		-
9.0		-

**Table A3:** The distance for axial and lateral resolution in mm for depth of 14.0, 16.0, 18.0 and 24.0 cm depth for curve probe at surface 4.

Depth (cm)	Diagram	Distance (mm)
14.0	Axial resolution	D1 = 4.9 D2 = 4.2
	Lateral resolution	D1 = 5.0
16.0	Axial resolution	D1 = 5.6 D2 = 4.1 D3 = 3.0
	Lateral resolution	D1 = 5.0
18.0	Axial resolution	D1 = 5.0 D2 = 4.1 D3 = 3.1
	Lateral resolution	D1 = 5.0
24.0	Axial resolution	D1 = 5.0 D2 = 4.1
	Lateral resolution	D1 = 4.8

**Table A4:** The distance for axial and lateral resolution in mm for depth of 5.0, 6.0, 7.0 and 9.0 cm depth for flat probe at surface 4.

Depth (cm)	Diagram	Distance (mm)
5.0	The horizontal distance cannot be measured for the image of this depth	-
6.0		-
7.0		-
9.0		-

## Appendix B

**Table A5:** The image taken for focal zone determination in different depth for curve probe.

Depth (cm)	Diagram
14.0	
16.0	
18.0	
24.0	

**Table A6:** The image taken for focal zone determination in different depth for linear probe.

Depth (cm)	Diagram
5.0	The horizontal distance cannot be measured for the image of this depth
6.0	The horizontal distance cannot be measured for the image of this depth
7.0	
9.0	

**Table A7:** The image taken for focal zone determination in different depth using linear probe on phantom 2.

Depth (cm)	Diagram
5.0	
6.0	
7.0	
9.0	

## References

[1] Ortiz S.H.C., Chiu T., Fox M.D. (2012). Ultrasound image enhancement: A review. Biomed. Signal Process. Control.

[2] Lawrence J.P. (2007). Physics and instrumentation of ultrasound. Crit. Care Med..

[3] Gan T., Hutchins D., Billson D., Schindel D. (2001). The use of broadband acoustic transducers and pulse-compression techniques for air-coupled ultrasonic imaging. Ultrasonics.

[4] Freudenrich C. (2001). How ultrasound works. How Stuff Work.

[5] Kessler C., Bhandarkar S. (2010). Ultrasound training for medical students and internal medicine residents—A needs assessment. J. Clin. Ultrasound.

[6] Noguchi S., Kobayashi T. (2022). Ultrasound Viscoelastic Properties of Biomass Polysaccharide Hydrogels as Evaluated by Rheometer Equipped with Sono-Device. Gels.

[7] Song T., Xiong Z., Shi T., Monto A.R., Yuan L., Gao R.J.G. (2021). Novel Fabrication of Zein-Soluble Soybean Polysaccharide Nanocomposites Induced by Multifrequency Ultrasound, and Their Roles on Microstructure, Rheological Properties and Stability of Pickering Emulsions. Gels.

[8] Sakalauskas A., Jurkonis R., Gelman S., Lukoševičius A., Kupčinskas L. (2019). Investigation of Radiofrequency Ultrasound-Based Fibrotic Tissue Strain Imaging Method Employing Endogenous Motion. J. Ultrasound Med..

[9] Oglat A.A., Alshipli M., Sayah M.A., Ahmad M.S. (2020). Artifacts in Diagnostic Ultrasonography.

[10] Shalbi S.M., Oglat A.A., Albarbar B., Elkut F., Qaeed M., Arra A.A. (2020). A brief review for common doppler ultrasound flow phantoms. J. Med. Ultrasound.

[11] Ahmad M.S., Suardi N., Shukri A., Ab Razak N.N.A.N., Oglat A.A., Makhamrah O., Mohammad H. (2020). Dynamic Hepatocellular Carcinoma Model Within a Liver Phantom for Multimodality Imaging. Eur. J. Radiol. Open.

[12] Afzal S., Zahid M., Rehan Z.A., Shakir H.M.F., Javed H., Aljohani M.M.H., Mustafa S.K., Ahmad M., Hassan M.M. (2022). Preparation and Evaluation of Polymer-Based Ultrasound Gel and Its Application in Ultrasonography. Gels.

[13] Himabindu M., Palanisamy A. (2017). Ultrasound- and Temperature-Induced Gelation of Gluconosemicarbazide Gelator in DMSO and Water Mixtures. Gels.

[14] Miller D.L., Smith N.B., Bailey M.R., Czarnota G.J., Hynynen K., Makin I.R.S., Bioeffects Committee of the American Institute of Ultrasound in Medicine (2012). Overview of therapeutic ultrasound applications and safety considerations. J. Ultrasound Med..

[15] Khanal S.K., Grewell D., Sung S., Van Leeuwen J. (2007). Ultrasound applications in wastewater sludge pretreatment: A review. Crit. Rev. Environ. Sci. Technol..

[16] Ammar A.O., Matjafri M., Suardi N., Oqlat M.A., Oqlat A.A., Abdelrahman M.A., Farhat O., Ahmad M.S., Alkhateb B.N., Gemanam S.J. (2018). Characterization and Construction of a Robust and Elastic Wall-Less Flow Phantom for High Pressure Flow Rate Using Doppler Ultrasound Applications. Nat. Eng. Sci..

[17] Wagner J.M., Monfore N., McCullough A.J., Zhao L., Conrad R.D., Krempl G.A., Alleman A.M. (2019). Ultrasound-Guided Fine-Needle Aspiration with Optional Core Needle Biopsy of Head and Neck Lymph Nodes and Masses: Comparison of Diagnostic Performance in Treated Squamous Cell Cancer Versus All Other Lesions. J. Ultrasound Med..

[18] Barnett S., Maulik D. (2001). Guidelines and recommendations for safe use of Doppler ultrasound in perinatal applications. J. Matern. Fetal Med..

[19] Mirza W.A., Imam S.H., Mohd Salim K., Aslam M., Ali S.A., Masroor I., Ahmad M.N. (2008). Cleaning methods for ultrasound probes. J. Coll. Physicians Surg. Pak..

[20] Koibuchi H., Kotani K., Taniguchi N. (2013). Ultrasound probes as a possible vector of bacterial transmission. Med. Ultrason..

[21] Oglat A.A., Matjafri M., Suardi N., Oqlat M.A., Abdelrahman M.A., Oqlat A.A. (2018). A review of medical doppler ultrasonography of blood flow in general and especially in common carotid artery. J. Med. Ultrasound.

[22] Oglat A.A., Suardi N., Matjafri M., Oqlat M.A., Abdelrahman M.A., Oqlat A.A. (2018). A review of suspension-scattered particles used in blood-mimicking fluid for Doppler ultrasound imaging. J. Med. Ultrasound.

[23] Oglat A.A., Matjafri M., Suardi N., Abdelrahman M.A., Oqlat M.A., Oqlat A.A. (2018). A new scatter particle and mixture fluid for preparing blood mimicking fluid for wall-less flow phantom. J. Med. Ultrasound.

[24] Oglat A.A., Matjafri M., Suardi N., Abdelrahman M.A., Oqlat M.A., Oqlat A.A., Abdalrheem R., Almutairi A. (2018). Acoustical and physical characteristic of a new blood mimicking fluid phantom. J. Phys. Conf. Ser..

[25] Alshipli M., Sayah M.A., Oglat A.A. (2020). Compatibility and validation of a recent developed artificial blood through the vascular phantom using doppler ultrasound color-and motion-mode techniques. J. Med. Ultrasound.

[26] Dakok K., Matjafri M., Suardi N., Oglat A.A., Nabasu S.E. (2022). Determination of the effect of glucose and cholesterol on the flow velocity of Blood Mimicking Fluid in a Common Carotid Artery Wall-less Phantom. J. Posit. Sch. Psychol..

[27] Dakok K., Matjafri M., Suardi N., Oglat A.A., Sirisena U.A. (2022). Influences of Body Composition on Carotid Artery Structure and function in Adult Humans. J. Posit. Sch. Psychol..

[28] Fatemi M., Greenleaf J.F. (2000). Probing the dynamics of tissue at low frequencies with the radiation force of ultrasound. Phys. Med. Biol..

[29] Weng L., Perozek D.M., Zhang J. (2004). Ultrasound Transducers for Imaging and Therapy. U.S. Patent.

[30] Maruvada S., Harris G.R., Herman B.A., King R.L. (2007). Acoustic power calibration of high-intensity focused ultrasound transducers using a radiation force technique. J. Acoust. Soc. Am..

[31] Oglat A.A., Matjafri M., Suardi N., Oqlat M.A., Abdelrahman M.A., Oqlat A.A., Abdalrheem R. (2018). Measuring the acoustical properties of fluids and solid materials via dealing with a-SCAN (GAMPT) Ultrasonic. J. Phys. Conf. Ser..

[32] Kulkarni N.M., Griffin M.O., O’Connor S.D., Sudakoff G. (2019). Sonographic Appearances of Portal Vein Abnormalities. Ultrasound Q..

[33] Maller V.V., Cohen H.L. (2019). Neonatal Head Ultrasound: A Review and Update—Part 1: Techniques and Evaluation of the Premature Neonate. Ultrasound Q..

[34] Oglat A.A., Dheyab M.A. (2021). Performance evaluation of ultrasonic imaging system (Part I). J. Med. Ultrasound.

[35] Dakok K.K., Matjafri M.Z., Suardi N., Oglat A.A., Nabasu S.E. (2021). A blood-mimicking fluid with cholesterol as scatter particles for wall-less carotid artery phantom applications. J. Ultrason..

[36] Dakok K.K., Matjafri M.Z., Suardi N., Oglat A.A., Nabasu S.E. (2021). A review of carotid artery phantoms for doppler ultrasound applications. J. Med. Ultrasound.

[37] Athamnah S.I., Oglat A.A., Fohely F. (2021). Diagnostice breast elastography estimation from doppler imaging using central difference (CD) and least-squares (LS) algorithms. Biomed. Signal Process. Control.

[38] Kim P.N., Lim J.W., Kim H.C., Yoon Y.C., Sung D.J., Moon M.H., Kim J.S., Kim J.C. (2008). Quality assessment of ultrasonographic equipment using an ATS-539 multipurpose phantom. J. Korean Radiol. Soc..

[39] Wang J., Lu J.-Y. (2007). Effects of phase aberration and noise on extended high frame rate imaging. Ultrason. Imaging.

[40] Park J.H., Heo Y.C., Han D.K. (2021). A Study on the Quality Control of Transvaginal Ultrasound Transducer using ATS-539 Ultrasound Phantom. J. Korean Soc. Radiol..

[41] Narayana P., Ophir J. (1983). A closed form method for the measurement of attenuation in nonlinearly dispersive media. Ultrason. Imaging.

[42] Wu W.-T., Chang K.-V., Hsu Y.-C., Hsu P.-C., Ricci V., Özçakar L.J.D. (2020). Artifacts in musculoskeletal ultrasonography: From physics to clinics. Diagnostics.

